# Ionizing air affects influenza virus infectivity and prevents airborne-transmission

**DOI:** 10.1038/srep11431

**Published:** 2015-06-23

**Authors:** Marie Hagbom, Johan Nordgren, Rolf Nybom, Kjell-Olof Hedlund, Hans Wigzell, Lennart Svensson

**Affiliations:** 1 Division of Molecular Virology, Department of Clinical and Experimental Medicine, University of Linköping, 581 85 Linköping, Sweden; 2 Department of Microbiology, Karolinska Institute, Stockholm, Sweden; 3 Department of Diagnostics and Vaccine, Swedish Institute for Communicable disease Control, Stockholm, Sweden

## Abstract

By the use of a modified ionizer device we describe effective prevention of airborne transmitted influenza A (strain Panama 99) virus infection between animals and inactivation of virus (>97%). Active ionizer prevented 100% (4/4) of guinea pigs from infection. Moreover, the device effectively captured airborne transmitted calicivirus, rotavirus and influenza virus, with recovery rates up to 21% after 40 min in a 19 m^3^ room. The ionizer generates negative ions, rendering airborne particles/aerosol droplets negatively charged and electrostatically attracts them to a positively charged collector plate. Trapped viruses are then identified by reverse transcription quantitative real-time PCR. The device enables unique possibilities for rapid and simple removal of virus from air and offers possibilities to simultaneously identify and prevent airborne transmission of viruses.

There is an urgent need for simple, portable and sensitive devices to collect, eliminate and identify viruses from air, to rapidly detect and prevent outbreaks and spread of infectious diseases^1^. Each year, infectious diseases cause millions of deaths around the world and many of the most common infectious pathogens are spread by droplets or aerosols caused by cough, sneeze, vomiting etc.^2^,^3^,^4^,^5^. Knowledge of aerosol transmission mechanisms are limited for most pathogens, although spread by air is an important transmission route for many pathogens including viruses^6^.

Today no simple validated technology exists which can rapidly and easily collect viruses from air and identify them. The problem is not the analyzing technique, since molecular biological methods such as real-time PCR enable a sensitive detection system of most pathogens^7^,^8^,^9^. The difficulty is to develop an effective sampling method to rapidly collect small airborne particles including viruses from large volumes of air. Furthermore, the sampling method should be robust with easy handling to enable a wide distribution and application in many types of environment. At present, the most commonly used techniques aimed to collect pathogens from air are airflow and liquid models^10^,^11^,^12^,^13^,^14^,^15^. These systems are complex, and their efficiency has not been thoroughly evaluated.

Spread of infectious diseases in hospitals can be most significant^16^,^17^,^18^. In many situations there is a need for a pathogen- and particle-free environment, e.g. in operation wards, environments for immunosuppressed patients as well as for patients with serious allergies. This makes it desirable to have a method not only for collection and identification^19^, but also for eliminating virus and other pathogens from air^20^. Ozone gas has been shown to inactivate norovirus and may be used in empty rooms to decontaminate surfaces, however in rooms with patients ozone should not been used due to its toxicity^21^. Generation of negative ions has previously been shown to reduce transmission of Newcastle disease virus^22^,^23^ and several kind of bacteria^24^,^25^ in animal experimental set-ups.

The ionizing device used in this study operates at 12 V and generates negative ionizations in an electric field, which collide with and charge the aerosol particles. Those are then captured by a positively charged collector plate. For safety reasons, the collector plate has a very low current, less than 80μA, however the ionizer accelerates a voltage of more than 200,000 eV, which enables high production of several billion electrons per second. Moreover, this device does not produce detectable levels of ozone and can thus be safely used in all environments.

This technique is known to effectively collect and eliminate cat-allergens from air^26^. Aerosolized rotavirus, calicivirus and influenza virus particles exposed to the ionizing device were attracted to the collector plate and subsequently identified by electron microscopy and reverse transcription quantitative real-time PCR techniques. Most importantly, we demonstrate that this technology can be used to prevent airborne-transmitted influenza virus infections.

## Results

### Visualization and efficiency of aerosol sampling as determined by electron microscopy

To develop and validate the ionizing technique for collection and identification of viral pathogens, we used several viruses of clinical importance; calicivirus, rotavirus and influenza virus (H3N2, strain Salomon Island) as well as latex particles. Canine calicivirus (CaCV, strain 48) was used as a surrogate^27^ for human norovirus, the aetiological agent behind the “winter vomiting disease”, causing outbreaks of great clinical and economic importance^28^. Rhesus rotavirus was used as a surrogate marker for human rotavirus^29^.

**Figure 1:**
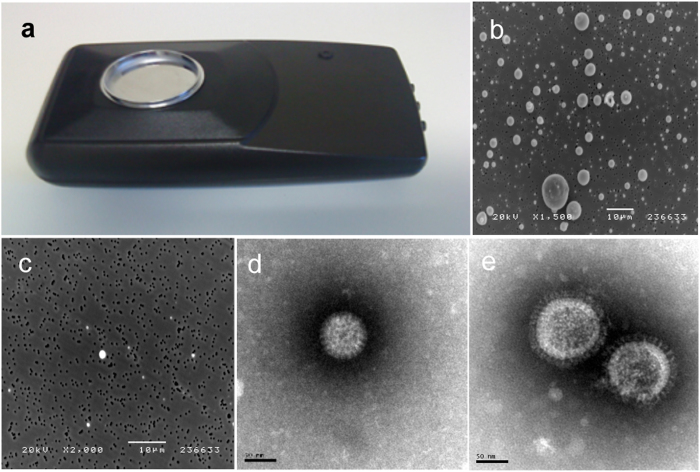
Airpoint ionizer with collector plate (size 13 × 35 cm) (**a**). The ionizing device was developed based of the Ion-Flow Ionizing Technology from LightAir AB, Solna, Sweden and was modified by installing a plastic-cup with a conductive surface of 47 mm in diameter, with positive charge, as the collector plate; Aerosolized and trapped latex particles (>1 to <10 μm) on active (**b**) and inactive (**c**) ionizer, (bar = 10 μM); Rotavirus (**d**); and influenza virus (H1N1; strain Salomon Island) (**e**) trapped on active ionizer, (Bar = 50 nm).

The device (Fig. 1a) consists of a small portable 12 volt operated ionizer, with a collector plate of positive charge attached to the ionizer, attracting negative particles from the air by electrostatic attraction. To determine optimal time collection parameters, latex particles with sizes ranging from <1 to >10 μm were nebulized into a room of 19 m^3^. Testing revealed that 40–60 min was required to eliminate >90% of free latex particles in the air as determined by real-time particle counting (PortaCount Plus). The particle counter can detect particles with size greater than 0.02 μM. Visualization by scanning electron microscopy (SEM) on grids from active- and inactive ionizer collector plates showed that accumulation of latex particles was dramatically enhanced on active ionizer collector plates compared to the inactive (Fig. 1b,c). Next, high numbers of rotavirus and formalin-inactivated influenza virus were aerosolized under the same conditions. While, after 40 min the inactive collector plates contained few (<5) rotavirus and influenza virus, the active collector contained >50 virus particles, as determined by transmission electron microscopy (TEM), (Fig. 1d,e).

### Ionizing air and electrostatic attraction collects aerosol-distributed viruses as determined by RT-qPCR

**Figure 2:**
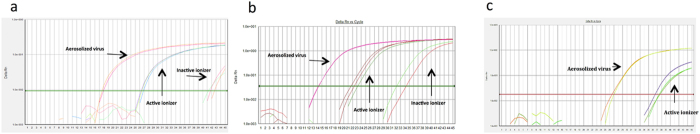
Real-time PCR on trapped rotavirus (**a**), calicivirus (**b**) and influenza virus (H1N1; strain Salomon Island) (**c**). Note that no influenza virus was detected on the inactive ionizer.

We next determined the capacity of RT-qPCR technology to quantitate the capacity of the ionizer technique to collect and concentrate viruses. Three independent experiments with each of the three viruses were carried out using the same virus concentrations in each experiment (Fig. 2a–c). Although several steps are involved from collection to detection the system was robust as to reproducibility. The RT-qPCR data shows that the active collector is concentrating and collecting virus 1500–3000 times more efficient as compared to the inactive collector (Table 1). When different dilutions of virus was used for aerosol production the proportion of aerosolized virus collected on the active collector was normally in the range of 0.1–0.6% for CaCV, rotavirus and influenza virus. A reproducible finding with regard to CaCV was a significant increase in relative recovery at the lowest concentrations increasing to 10–20% of the total amount of virus aerosolized (Table 1).

### Ionizing air reduces calicivirus and rotavirus infectivity

Next we determined if collected viruses retained their infectivity after being exposed to negative ions and/or after being exposed to the positively charged collector plate. Five mL of cell culture medium (Eagles Minimal Essential Media (Eagles MEM)) containing 1 × 10^6^ peroxidase forming units of rotavirus respectively of CaCV were aerosolized and collected during 40 min to an active collector plate, containing 1 mL of Eagles MEM. CaCV in cell culture medium was also directly exposed to an active and inactive collector plate, without being aerosolized. Viral infectivity was determined essentially as described^30^ and the ratio between viral genome copy numbers versus infectivity was compared between aerosolized virus, virus exposed to active- and inactive collector plates and the viral stocks. CaCV exposed to an active collector plate, without being aerosolized, showed a slight reduction in infectivity (~40%) in comparison to virus that have been trapped on an inactive collector plate (Table 2). In contrast, the infectivity of aerosolized viruses was greatly reduced by >97%, indicating that ionization of the aerosol accounts for the vast majority of infectivity reduction, and not the exposure to the charged collector plate.

Further support that ionizing was the mechanism by which viruses lost infectivity comes from experiments were rotavirus was nebulized without ionizing and allowed to be trapped to an inactive collector plate. Collectors were located at 30 cm from the nebulizer. The result concluded that the genome copy versus infectivity ratio was unchanged from that of the viral stock, thus suggesting that inactivation of virus is associated with ionized air.

### Ionizing air and electrostatic attraction prevents airborne-transmitted influenza A/Panama virus infection between guinea pigs

**Figure 3:**
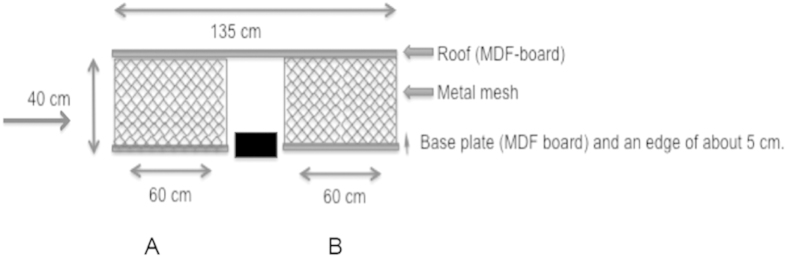
Set-up design of influenza virus (H3N2, Pan/99) aerosol-transmission experiments between guinea pigs. Guinea pigs (n = 4) were intranasally infected with 5 × 10^3^ pfu of Pan/99 virus in 100 uL (50 uL in each nostril). All four infected animals were placed into an experimental cage “A”. At 30 h p.i. four naïve uninfected guinea pigs were placed in cage “B” . Air-flow from left to right. Air exchanged 17x/day. Filled rectangle = ionizer.

Next we took advantage of an established influenza guinea pig model^31^,^32^,^33^ to study if ionizing air and electrostatic attraction could prevent airborne aerosol and droplet transmitted influenza A/Panama (Pan/99) virus infection between guinea pigs. The airborne/droplet transmission model was established essentially as described^31^ using two separate cages with the ionizer placed between the cages (Fig. 3). Four guinea pigs were infected by intranasal route as described with 5 ×10^3^ pfu of Pan/99^31^ and placed in cage “A” (Fig. 3). At 30 hours post infection (h p.i.) 4 uninfected guinea pigs were placed in cage “B” 15 cm from the cage with infected animals as illustrated in Fig. 3, with no physical contact. The ionizer was placed between cages “A” and “B”. Two identical experiments were performed, one with active ionizer placed between the cages and one with an inactive ionizer.

Uninfected animals in cage “B” were exposed for 24 hours with airflow from cage “A” hosting the 4 infected guinea pigs and then placed in individually ventilated cages for the next 21 days, to ensure that the only time-point for being infected was the 24 hours when they were exposed to air from infected animals in cage “A”. RT-qPCR of lung- and trachea biopsies examined at 54 h p.i. from the nasally experimentally infected animals, revealed that 3 out of 4 guinea pigs in both experiments were positive for influenza.

**Figure 4:**
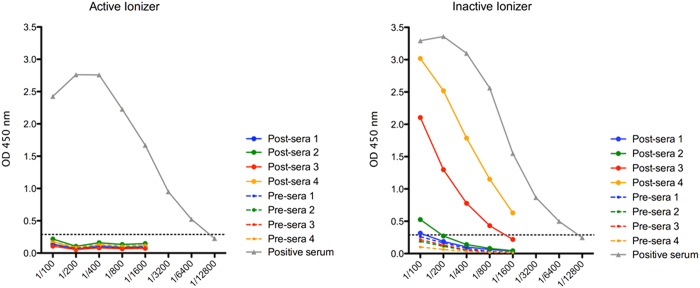
Active ionizer prevents aerosol transmitted influenza virus (H3N2, Pan/99) infection between guinea pigs. While the active ionizer prevented 4 of 4 exposed guinea pigs from developing an immune response to influenza virus, 3 of 4 animals were infected when the inactive ionizer was used. Graph shows antibody titers by ELISA before infection (pre-serum 1, 2, 3 and 4) and at day 21 post-exposure to influenza virus (post-serum 1, 2, 3 and 4). Briefly, influenza virus H1N1; (SBL Influenza Vaccine, Sanofi Pasteur, Lyon, France) were coated on ELISA plates and incubated with two-fold dilutions of pre- and post- guinea pig sera, followed by biotinylated rabbit-anti-guinea pig antibody, HRP conjugated streptavidin and TMB substrate as described in Methods. Cut off (dashed line) value (0.284 OD) was the mean of the negative controls +2SD.

We assessed transmission of infection from animals in cage “A” to exposed uninfected animals in cage “B” by development of an immune response 21 days post exposure. The results shown in Fig. 4 illustrate that when the ionizer was inactive, 3 of the 4 uninfected but exposed animals developed a serum IgG influenza-specific immune responses. In contrast, none of the 4 animals in cage “B” developed an immune response to influenza virus when the ionizer was active (Fig. 4). Furthermore, influenza virus RNA could be detected by RT-qPCR, albeit at low concentration, on the collector plate from the active ionizer but not with the inactive ionizer, showing that the ionizing device indeed collected virus excreted from the infected animals in cage “A”.

## Discussion

We describe a simple ionizing device operating at 12 volt that can prevent spread of airborne transmitted viral infections between animals in a controlled setting, whilst simultaneously collecting virus from air for rapid identification. Coupled with sensitive RT-qPCR assays, this sampling method enabled fast detection and highly sensitive quantification of several human clinically important viruses such as influenza virus, rotavirus and calicivirus. The device consists of a small portable ionizer, where a sampling cup of positive charge is attached to the ionizer attracting negative particles from the air. Important advantages with this novel ionizing device is the simple handling, high robustness as well as the wide applicability to airborne pathogens.

The observation that significantly higher numbers of rotavirus and CaCV particles were detected on the active ionizer compared to the inactive ionizer (~1500–3000 times), led to the conclusion that this technique can actively and efficiently collect viral particles from air. Similarly, visualization of latex particles by SEM revealed that latex particles of all sizes investigated were concentrated on the active collector. It is interesting to note that a broad range of particles sizes, from 35 nm to 10 μm was concentrated, suggesting a wide application range of the technology. However, too large particles may decrease the recovery since these are proposed to remain for less time in the air^33^,^34^.

Interestingly, when we aerosolized low amounts of CaCV, (1.56 × 10^4^ gene copies and 1.87 × 10^3^ gene copies), we observed collection recoveries of 10.6 and 21%, respectively. This markedly increased efficiency, with smaller amounts of virus distribution in air, could be due to less aggregation of virus-virus or virus-cell debris particles more long lasting airborne, and thus leads to stronger electrostatic attraction by the collector. Furthermore, it is likely that much particles end up at the walls of the collector plate or on areas adjacent to the collector plate on the ionizer; and are subsequently not quantified by real-time PCR; thus underestimating the electrostatic effect. When aerolizing higher virus concentrations, this effect can thus lead to lower estimates of recovery. Using CaCV, rotavirus and influenza virus, we performed three independent experiments for each concentration of aerosolized virus in order to assess the robustness of the assay throughout all steps (collection with active ionizer, RNA extraction, cDNA synthesis and real-time PCR). Although several steps are involved from collection to detection we found the assay to be highly robust since the minimum and maximum quantity of virus from each independent measurement was always within a range of 1 log (Fig. 2).

Inactivation of viruses by electrostatic attraction has only been briefly investigated^35^. In the present study, rotavirus and CaCV lost significant (>97%) infectivity (ratio; CaCV from 3.0 × 10^−2^ to <7.8 × 10^−4^ and rotavirus from 4.9 × 10^−1^ to <7.6 × 10^−3^) in ionized air as determined by a ratio of infectivity versus gene copies. The mechanism of inactivation was not explicitly investigated in this study, but inactivation mechanisms may include reactive species and/or increased protein charge levels, which could inactivate virus as previously described^36^,^37^. Reduced infectivity has been proposed to be due to reactive oxygen species and ozone, through lipid- and protein peroxidation reactions that may cause damage and destruction to the viral lipid envelope and protein capsid^36^. In particular, protein peroxidation may play a key role in the inactivation of non-enveloped viruses, such as adenovirus, poliovirus and other enteroviruses such as rota- and caliciviruses. Enveloped viruses are suggested to lose infectivity due to lipid peroxidation. However, the cytotoxity of ozone creates a major obstacle for the clinical application of ozone. It has been shown hat increasing the ion concentration of the air efficiently protect chickens from air-born transmission of lethal Newcastle disease virus infection^23^. The exact mechanism of negative ion inactivation of viruses has not been shown and needs to be further investigated. However, in a study using generation of negative and positive ions, influenza virus was inactivated although ozone level was negligible (0.005 ppm or less)^37^.

Our device released a steady-state ozone concentration below the detection limit (0.002 ppm) as tested by VTT (Technical Research Center of Finland, Tampere, Finland) and by Air Resources Board in the US, thus ozone cannot in this case be a contributor of viral inactivation. However, reactive radicals such as •O_2_^-^ may be generated, which may contribute to inactivation through damage to either the protein or the nucleic acid structure of the viruses^37^. As infectivity was not lost when virus was nebulized into the air of the room without ionization and only slightly reduced when applied directly on the positively charged collector plate, it is suggested that most reduction of infectivity may be due to increased negative charged levels, presumably resulting in changes in isoelectric point and thus structural changes of the capsid. As the two viruses investigated are non-enveloped, lipid modification can be ruled out.

Most interesting, and of great clinical significance of this study was the novel finding that the ionizing device could detect and prevent influenza virus infection in a controlled setting, mimicking “authentic” conditions. Our intranasal infection protocol was essentially as previously described^31^,^33^ using Harley guinea pigs and 5 × 10^3^ plaque forming units (pfu) of Pan/99 influenza virus. As guinea pigs of the Hartley strain are highly susceptible to human influenza A virus strain Pan/99 (H3N2), with an infectious dose (ID_50_) of 5 pfu^31^, this makes the viral strain most appropriate for these studies. Moreover, Lowen and co-workers have shown 100% transmission of Pan/99 by aerosol to guinea pigs^38^,^39^. Previous studies have also shown that the used infectious dose results in a viral growth peak around day 3 p.i. in both lungs and nasal passages in this animal model^31^, a time point when naïve animals in our study was exposed to air from the infected animals.

We found, by assessing development of the immune response, that 3 of 4 uninfected guinea pigs became infected after exposure to animals inoculated with 5 × 10^3^ pfu of Pan/99. These susceptibility figures are similar to those of Mubareka and co-workers^33^ who found that 2 of 3 guinea pigs became infected following short-range aerosol transmission with a dose of 10^3^ pfu whereas 3 of 3 animals became infected with a infectious dose of 10^6^ pfu. We examined the immune response at 21 days p.i., a time point when Lowen and co-workers^40^ previously have found that naturally Pan/99 infected guinea pigs had developed a significant immune response.

The mode of influenza virus transmission includes direct contact with individuals, exposure to virus-contaminated objects (fomites) and inhalation of infectious aerosols. Previous studies using the guinea pig animal model have indicated that aerosol and not fomites is the principal route of Pan/99 transmission between guinea pigs^33^. Aerosol released virus from inoculated animals could be detected on the active collector plate by RT-qPCR, albeit at very low gene copy numbers. Using the guinea pig as a host model, Lowen *et al.*^38^ showed that aerosol spread of influenza virus between animals is dependent upon both the relative humidity and temperature. They found that low relative humidity of 20–30% and temperature of 5 °C was most favourable, with no transmission detected at 30 °C. In our set-up system, the temperature was between 20–21 °C and relative humidity between 35–36.2%.

The described ionizing device coupled with RT-qPCR assays has a clear diagnostic potential. The easy handling, low cost, free of ozone production, robustness, high efficiency and low-voltage (12 volt) operation enables large-scale use. Locations critical for infectious spread, such as airplanes, hospitals, day-care centres, school environments and other public places could thus be monitored and controlled by the collection and analysis of airborne viruses and other pathogens on the collector plate. The device also show potential for transmission prevention, although the potency needs to be further investigated in real-life settings. We conclude that this innovative technology hold great potential to collect and identify viruses in environmental air.

## Methods

### Study design

The experimental room has grounded metal walls, with a volume of 19 m^3^ (B250*L330*H235cm). One active and one inactive ionizer device, designed for collection and analysis of particles in the air, were placed in the room at equal distance (215 cm) from the nebulizer (Aiolos Albatross, Aiolos, Sweden), with a distance of 64 cm between the ionizers. A particle counter (PortaCount Plus, TSI Incorporated, USA) was used before and during the experiment. Before the start of aerosol experiments, the room was emptied on particles by the active ionizer and the collector plate was discarded before the experiments begun and replaced with a new collector plate. The experiments were continued until the particle counts were back to basal level, usually reached within 40 min. Humidity and temperature conditions were measured initial to each aerosol experiments.

### Ionizer technology and device

The ionizing device used in this study was developed on the basis of the ion-flow ionizing technology from LightAir AB, Solna, Sweden (www.lightair.com) and was modified for this work by the Department of Microbiology, Karolinska Institute, Stockholm, Sweden. The device (size of 13 × 35 cm) was modified by installing a plastic-cup with a conductive surface of 47 mm in diameter (GP plastindustri, Gislaved, Sweden) as the collector plate (Fig. 1). The collector plate has for safety reasons a very low current, less than 80 μA, however the ionizer accelerates an extremely high voltage of more than 200,000 eV. The ionizer creates electrons, which will render surface molecules of particles in air negatively charged thus attracting them to the positively charged collector plate. This device generates approximately 35 000 billion electrons per second (www.lightair.com) with a steady-state ozone concentration below the detection limit (0,002 ppm) as tested by VTT Technical Research Center of Finland, Tampere, Finland. It has also been ozone tested and certified by ARB (Air Resources Board) in the US. After the end of the sampling period, the ionizer was turned off, and the collector plate was covered with a lid and stored at −20 °C until analysis. Viruses captured on the collector plates were analyzed by a RT-qPCR for rotavirus, CaCV and influenza virus, and the results from the active- and inactive ionizers were compared. Scanning- and transmission electron microscopy were used for visualization of collected viruses and latex-particles.

### Aerosol experiments of virus and latex particles

Different amounts of rhesus rotavirus (genotype G3P[3]), influenza virus (strain H1N1, Salomon Island, inactivated) and CaCV strain 48 (genus *Vesivirus*) were diluted in water to a final volume of 5 mL. In aerosol experiments for scanning electron microscopy and infectivity analysis, virus was diluted in Eagles MEM. Virus suspensions in different concentrations were distributed as aerosols in the room by the use of a nebulizer. Each experiment was performed in triplicates and collection of aerosolized virus and latex particles were performed during 40 min.

### Transmission electron microscopy (TEM)

Carbon/Formvar-coated 400 mesh copper grids were placed on the collector during aerosol experiment with influenza- and rotavirus. Grids were then rehydrated in Eagles MEM containing 1% bovine serum albumin (BSA) before being negatively stained with 2% phosphotungstic acid and analyzed by TEM. Ten grid squares were analyzed per specimen and the number of virus particles per unit area was calculated.

### Scanning electron microscopy (SEM)

Collected samples were added on the surface of a polycarbonate 0.6 μm filter (Nucleopore, Inc) which was fitted to an airtight gadget (GP Plastindustri AB, Gislaved, Sweden). The filter was dried in room temperature, coated with 40 Å thick layer of ionized gold and analyzed by SEM (Philips High Resolution SEM 515). The method has previously been used and reported in studies of cytomegalovirus as well as cerebrospinal fluid^41^,^42^,^43^.

### Extraction of viral RNA from collector plates

The attached viral particles on the collector plates were lyzed with 1 mL of viral lysis buffer (buffer AVL, QIAamp viral RNA mini kit) added directly into the collector plate and immediately proceeded for extraction of viral RNA using QIAamp Viral RNA Mini Kit (Cat.no: 52906 Qiagen, Hilden, Germany) according to the manufacturer’s instructions. Each sample was eluted with 60 μL of RNase-free water containing 0.04% sodium azide (AVE buffer; Qiagen, Hilden, Germany).

### Reversed transcription of extracted viral RNA

28 μL of the extracted viral RNA was mixed with 50 pmol of Pd(N)_6_ random hexamer primer (GE-Healthcare, Uppsala, Sweden) and quickly chilled on ice for 2 min, followed by the addition of one Illustra Ready-To-Go reverse transcriptase PCR (RT-PCR) bead (GE-Healthcare, Uppsala, Sweden) and RNase-free water to a final volume of 50 μL. For rotavirus, an initially denaturation step at 97 °C for 5 min was performed to denature the dsRNA. The reverse transcription (RT) reaction was carried out for 40 min at 42 °C to produce the cDNA later used for real-time PCR.

### Quantitative real-time PCR for rotavirus

Rhesus rotavirus was detected and quantified using a LUX real-time PCR assay as described previously^7^. This real-time PCR uses labeled primers with different fluorophores for each VP6 subgroup and external plasmid standards for semi-quantification^44^.

### Quantitative real-time PCR for CaCV

CaCV was detected and quantified using a SYBR green assay on a ABI prism 7500 (Applied Biosystems, Foster City, CA) with primers; (final concentration 200 nM) CaCV-3 (5-ACCAACGGAGGATTGCCATC-3´ (nucleotides 393 to 410 according to GenBank accession no. AF053720) and CaCV-4 (5´-TAGCCGATCCCACAAGAAGACA-3´ (nucleotides 452 to 474), specific for CaCV strain 48. The reaction was performed with 2 μL cDNA in 10 μL 2X SYBR Green PCR Master Mix (Applied Biosystems) and water to a final volume of 20 μL. The following cycling program was used: 95 °C for 10 min followed by 45 cycles of 95 °C for 15 seconds and 60 °C for 1 min. Melting curve analysis was performed immediately after PCR completion, by heating at 95 °C for 15 seconds, followed by cooling to 60 °C for 1 min and subsequent heating to 95 °C at 0.8 °C min^−1^ with continuous fluorescence recording. Melting temperatures were determined on all samples using the Sequence Detection Software version 1.3.1 (Applied Biosystems) and visualized by plotting the negative derivatives against temperature.

### Sampling for infectivity studies with rotavirus and CaCV

To determine whether the ionizing technology has any influence on virus infectivity, rhesus rotavirus and CaCV was aerosolized and collected on active ionizer collector plates, covered with 1 mL of Eagles MEM. Rotavirus (1 × 10^6^ peroxidase forming units) and CaCV (1 × 10^6^ peroxidase forming units) was aerosolized, each in a total volume of 5 mL and collected for 40 min followed by determination of viral infectivity and number of genome copies.

To determine if ionized air *per se* had an effect on viral infectivity, rhesus rotavirus was aerosolized and captured on a collector plate containing 1 mL of Eagles MEM, without ionization, placed at a distance of 30 cm from the nebulizer.

To determine if electrostatic attraction of the collector plate affected viral infectivity, rotavirus (2 × 10^5^ peroxidase forming units) and CaCV (2 × 10^5^ peroxidase forming units) in 1 ml of Eagles MEM, were added to inactive and active collector plates for 40 min. The plates were subsequently stored at −20 °C until determination of viral infectivity and number of genome copies.

### Determination of rotavirus and CaCV infectivity

Rotavirus stock and samples were diluted 1:10 in Eagles MEM and subsequently diluted in two-fold dilutions. Determination of viral infectivity was performed as previously described on confluent Green monkey kidney cells (MA104) in 96-well plates^30^. CaCV infectivity was determined essentially as for rotavirus with the modification that samples were added to confluent Madin-Darby Canine Kidney (MDCK) cells in 48-well plates and infectivity determined as previously described^45^ and confirmed by RT-qPCR. To determine the reduction of infectivity, the ratio of viral genome copy numbers versus infectivity was compared between aerosolized virus, virus exposed to active- and inactive collector plates and the viral stock.

### Animals

Guinea pigs, strain Hartley, female, 300–350 g, were housed at Astrid Fagraeus Laboratory, Karolinska Institute, according to approved guidelines from the Board of Agriculture and the Council of Europes Convention on vertebrate animals used for scientific purpose. The experimental protocol was approved by the Animal Ethics Committee in Stockholm (Permit Number: N177/11).

### Airborne transmission of influenza virus

We use a guinea pig animal model to investigate whether the ionizing technique could prevent transmission of influenza virus infection, since this model have successfully been used as a model of aerosol transmission studies of influenza virus^31^,^33^. Human influenza A virus, strain Pan/99 (kindly provided by Peter Palese, New York, USA) was used since this strain has been shown to effectively replicates in the upper respiratory airways and effectively transmit by aerosols but not by fomites in guinea pigs. Female guinea pigs, 300–350 g, strain Hartley, were housed at Astrid Fagreaus Laboratorium, Solna, Stockholm (Ethical permission N177/11). Four animals were anesthetized by an intra peritoneal injection of ketamin (Ketalar el Ketaminol) 50 mg/kg and xylazin (Rompun) 5 mg/kg and infected intranasally with 5 × 10^3^ pfu of Pan/99 virus in 100 uL (50 uL in each nostril). All four infected animals were placed into the experimental cage (Fig. 3, cage “A”). At 30 h p.i., four naïve uninfected guinea pigs were placed next to the transmission cage (Fig. 3, cage “B”) at a distance of 15 cm. Air flowed freely between cages, but direct contact between inoculated and exposed animals was prohibited.

The four naïve guinea pigs were exposed for 24 hours and then put into separately individually ventilated cages, to ensure that no aerosol transmission occurred between the animals. Two identical experiments were performed, with an active and inactive ionizer. The nasally infected animals were removed after the exposure time and lung and trachea biopsies were collected (54 h p.i.) and investigated for influenza virus by RT-qPCR. At 21 days post exposure, serum was collected from the uninfected exposed guinea pigs and the prevalence of antibodies against influenza A virus was determined by ELISA. Sera taken before exposure to the infected guinea pigs (pre-sera), and 21 days days after exposure (post-sera) were analyzed from each animal.

### ELISA detection of influenza A antibodies

Briefly, 96-well plates (Nunc, 96 F MAXISORP, Roskilde, Denmark) where coated with formalin-inactivated Influenza A virus H1N1 (SBL Influenza Vaccine, Sanoil Pasteur, Lyon, France) diluted in coating buffer (0.05 M sodium carbonate buffer, pH 9.5–9.7) at 5 μg/mL and incubated at +4 °C over night. Wells were washed x3 (0.9% NaCl and 0.05% Tween-20) and blocked with 3% BSA in PBS buffer for 1 hour at 37 °C. Serum samples were diluted 1:100 and further in two-fold dilutions in dilution buffer (PBS containing 0.5% BSA and 0.05% Tween-20), and incubated for 90 min at 37 °C. Plates were then washed x5 and incubated for 60 min at 37 °C with secondary biotinylated goat-anti guinea pig antibody (Vector, BA-7000) and horseradish-peroxidase (HRP) conjugated Streptavidin (DAKO, Denmark, P0397), both at a dilution of 1:3000. Plates were then washed x5 and 100 μL of tetramethyl benzidine (TMB) substrate (Sigma Aldrich, T-0440-16) was added to each well, the reaction developed for 10 min and stopped by addition of 100 μL of 2 M H_2_SO_4_. Absorbance was measured at 450 nm in an ELISA reader (VersaMax, Molecular Devices). Cut off values were calculated as the average value of negative controls OD and 2 times the SD.

### Extraction of influenza RNA from guniea pig tissue

RNA was extracted from trachea and lung tissue of infected guinea pig. Briefly, 100–250 mg of tissue were homogenized with a tissue homogenizer and total RNA extracted with RNAeasy Midi Kit (Qiagen) according to the manufacturer’s instructions.

### Quantitative real-time PCR for influenza virus

To detect and quantify influenza A virus on the collector plates as well as in guinea pig tissue samples, we used a One-Step Taq Man real-time RT-PCR assay^46^ with primers F1-mxA (150 nM) (5´-AAGACCAATYCTGTCACCTCTGA-3´), F3-mxA (150 nM) (5´-CAAGACCAATCTTGTCACCTCT GAC-3´) and R1-mxA (900 nM) (5´-TCCTCGCTCACTGGGCA - 3´) and probes P1-Mx (110 nM) (5´-FAM-TTGTGTTCACGCTCACC–MGB–3´) and P2-Mx (110 nM) (5´-FAM-TTTGTATTCACGCTCACCG–MGB -3´), with the Rotor-Gene Probe RT-PCR Kit (Qiagen). The real-time PCR reaction was performed in a Corbett Rotor-Gene 6000 (Qiagen) with the following cycling protocol: 50 °C for 10 min, followed by 45 cycles of 95 °C for 5 seconds and 57 °C for 15 seconds.

## Additional Information

**How to cite this article**: Hagbom, M. *et al.* Ionizing air affects influenza virus infectivity and prevents airborne-transmission. *Sci. Rep.* **5**, 11431; doi: 10.1038/srep11431 (2015).

## Figures and Tables

**Table 1:**
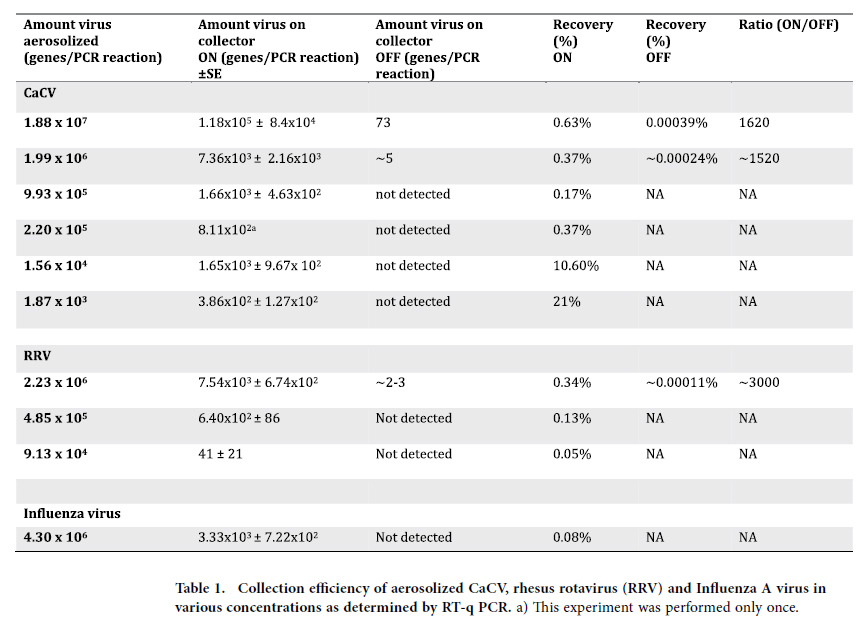
Collection efficiency of aerosolized CaCV, rhesus rotavirus (RRV) and Influenza A virus in various concentrations as determined by RT-q PCR.

**Table 2:** Reduction of infectivity of Canine Calicivirus (CaCV) and Rhesus Rotavirus (RRV).

	**Ratio of infectious virus particles to virus genes per PCR-reaction as quantified by RT-qPCR**					
	**Exposed to charged collector**	**Exposed to uncharged collector**	**Reduction of infectivity**	**Aerolized virus**	**Aerolized virus captured**	**Reduction of infectivity**
**CaCV**	0.74 × 10^−4^	1.24 × 10^−4^	40.1%	2.96 × 10^−2^	<7.83 × 10^−4^^a^	>97.4%^a^
**RRV**	n.d.	n.d.	n.d.	4.86 × 10^−1^	<7.66 × 10^−3^^a^	>98.4%^a^

^a^Under detection limit (10 peroxidase forming units/mL) on the infectivity assay.
